# A framework for reporting secondary and incidental findings in prenatal sequencing: When and for whom?

**DOI:** 10.1002/pd.6097

**Published:** 2022-01-19

**Authors:** Danya Vears, David J. Amor

**Affiliations:** ^1^ Murdoch Children's Research Institute Royal Children's Hospital Melbourne Victoria Australia; ^2^ Melbourne Law School University of Melbourne Carlton Victoria Australia; ^3^ Department of Paediatrics University of Melbourne Royal Children's Hospital Melbourne Victoria Australia

## Abstract

As the use of genomic sequencing (GS) in the prenatal setting becomes more widespread, laboratories and clinicians will be tasked with making decisions about whether to offer incidental and secondary findings to expectant parents and, if so, which ones. Unfortunately, few guidelines or position statements issued by professional bodies address the return of secondary findings specifically in the context of prenatal GS, nor do they offer clear guidance on whether, and which types of incidental findings should be reported. Laboratories and clinicians will also need to navigate other challenges, such as how to obtain sufficiently informed consent, workload burdens for both laboratories and clinicians, and funding. Here we discuss these, and other challenges associated with offering incidental and secondary findings in the context of prenatal GS. We outline existing guidelines for return of these findings, prenatally and in children. We review the existing literature on stakeholder perspectives on return of incidental and secondary findings and discuss the main practical and ethical challenges that require consideration. We then propose a framework to help guide decision‐making, suggesting a baseline routine analysis, with additional layers of analysis that could be offered, according to local laboratory policy, with additional opt‐in consent from the parents.

## Key points

What's already known about this topic?

- The use of genomic sequencing (GS) in the prenatal setting is becoming more widespread.
- Few guidelines or position statements from professional bodies address returning secondary findings in the context of prenatal GS.

What does this review add?

- We summarize existing guidelines and literature on the topic.
- We discuss practical and ethical challenges that require consideration.
- We propose a framework to guide decision‐making, suggesting baseline routine analysis, with additional layers of analysis that could be offered, with opt‐in parental consent.

## 1 INTRODUCTION

Genomic sequencing (GS) is rapidly being integrated into many areas of clinical care, including prenatal diagnosis. Along with the greater chance of identifying the cause of fetal abnormality compared to the previous gold standard of chromosomal microarray (CMA),^1^ GS has more potential to identify incidental (also known as unsolicited) findings.^2^ Incidental findings are variants in disease‐causing genes that are unrelated to the phenotype under investigation, and are identified by chance during the analysis.^3^ In contrast, secondary findings are variants in disease‐causing genes that are unrelated to the phenotype, but that are actively sought.^4^

Whether to return incidental findings and/or actively search for secondary findings has been discussed extensively. This debate was at its most heated in 2013 when the American College of Medical Genetics and Genomics (ACMG) published a position statement recommending that all samples undergoing diagnostic GS should be analyzed for a list of 56 genes, corresponding to a range of mainly hereditary cancer and cardiac conditions, and that pathogenic and likely pathogenic variants in these genes should be reported.^5^ Perhaps the most controversial aspects of this document were the suggestions that patients should not be able to opt out of such analysis (a suggestion that was subsequently revised),^6^ and that the policy applied to children as well as adults, despite some of the genes being associated with conditions with solely adult onset. Since then, the ‘ACMG list’ has been revised twice, in 2015^6^ and 2021,^7^ and now contains 73 genes.^8^

It is notable that while previously the ACMG stated that their recommendations do not apply to fetal samples,^5^ a recent ACMG ‘points to consider’ document on fetal exome sequencing suggests that secondary findings should be offered during pre‐test counselling,^9^ a point reinforced by their most recent guideline that the option to opt out of secondary findings should be presented in the context of prenatal sequencing.^7^ Yet, as we will show, few prenatal recommendations issued by professional bodies even address secondary findings in relation to prenatal GS, let alone offer clear guidance.

As use of GS in the prenatal setting becomes more widespread, laboratories and clinicians are going to be tasked with making decisions about whether to offer secondary findings to expectant parents and, if so, which ones. They will also need to navigate other challenges, such as how to obtain sufficiently informed consent, workload burdens for both laboratories and clinicians, and funding.

Herein we discuss these and other challenges associated with offering secondary findings in the context of prenatal GS. We outline existing guidelines for return of both incidental and secondary findings, prenatally and also in children. We review the existing literature on stakeholder perspectives on return of incidental and secondary findings and then propose a framework to help guide decision making.

## 2 REVIEWING THE GUIDELINES ON INCIDENTAL AND SECONDARY FINDINGS

### 2.1 Existing prenatal guidelines addressing incidental and secondary findings

Few position statements issued by professional bodies address the return of incidental or secondary findings in the context of GS,^10^, ^11^ and gaps in guidelines and recommendations have been identified.^12^ Where guidelines exist, only the Canadian College of Medical Geneticists (CCMG) and the ACMG provide clear guidance about whether, and which types of incidental findings should be reported.^9^, ^11^ Both agree that incidental findings that relate to pediatric‐onset conditions, and are pathogenic or likely pathogenic, should be reported regardless of whether they are medically actionable. The CCMG specifies that incidental findings relating to adult‐onset conditions should not be reported, regardless of the pathogenicity of the variant,^11^ whereas the ACMG frame this differently, recommending that variants with no known fetal or childhood phenotype should not be reported.^9^ Neither recommend reporting carrier status. Although the Joint Position Statement from the International Society for Prenatal Diagnosis (ISPD), the Society for Maternal Fetal Medicine (SMFM), and the Perinatal Quality Foundation (PQF) addresses incidental findings in the context of prenatal GS, they merely recognize that some laboratories may choose to report pathogenic and likely pathogenic variants in genes that lead to childhood‐ and adult‐onset conditions, and suggest this topic be covered during genetic counselling.^10^

Prenatal GS is commonly performed as a ‘trio’ design, comprising fetus and both parents, and therefore parental results also need to be considered. Regarding parental incidental findings, the ACMG propose laboratories need their own policies for whether parental incidental findings and carrier status are reported, and if so, recommend that these be limited to pathogenic and likely pathogenic variants.^9^ The CCMG recommends that reporting of parental incidental findings be limited to those identified in the fetus.^11^

Normative documents have discussed the return of incidental findings more comprehensively in the context of CMA.^13^, ^14^, ^15^ Of these, only two take a stance on whether incidental findings should be returned.^13^, ^15^ The position statement issued jointly by the Society of Obstetricians and Gynecologists of Canada and the CCMG states that incidental findings relating to highly penetrant, medically actionable, childhood‐onset conditions should be reported but that conditions that have adult‐onset or are non‐actionable should not be disclosed.^13^ Similarly, the recommendations by the UK‐based Royal College of Pathologists suggest that pathogenic findings that “will potentially inform the management of the pregnancy, or of the family, in the clinical context in which CMA was done or in the future, should be reported regardless of size of imbalance”.^15^

The Best Practice Guidelines from the Australasian Society of Diagnostic Genomics and Human Genetic Society of Australasia recommend that laboratories have their own procedures to deal with incidental findings,^14^ whereas the statement by the American College of Obstetricians and Gynecologists simply acknowledges that medically actionable incidental findings may be identified.^16^

Fewer normative documents address actively searching for secondary findings. The CCMG guidelines clearly state that laboratories should not actively search for variants unrelated to the reason for referral.^11^ In contrast, the ACMG recommends secondary findings be offered in the pre‐test counselling process, and reported if consent is obtained to do so.^9^ They suggest laboratories have their own policy regarding whether the secondary findings they report in the parents are limited to those identified in the fetus.^9^ The ISPD/SMFM/PQF Joint Position Statement does not make a recommendation about whether laboratories should search for secondary findings, but suggests that inclusion or exclusion of secondary findings should be discussed in the informed consent process.^10^

### 2.2 Existing guidelines addressing incidental and secondary findings children

Although the context is different, it is worth examining the normative documents addressing return of results from GS being conducted in children. Disagreement as to whether incidental findings and secondary findings should be reported for children exists between documents issued by different professional bodies. The documents that give clear guidance support the return of incidental and secondary findings for childhood‐onset, medically actionable conditions,^17^, ^18^ although the ACMG limits this to their curated list of genes.^5^ The European Society for Human Genetics (ESHG) does not provide clear direction, instead calling for guidelines to be established to address this.^19^ Differences between guidelines are more overt in relation to incidental findings for adult‐onset conditions. The ACMG's list contains numerous genes that can have onset in both adulthood and childhood, and a number that are purely adult‐onset conditions.^8^ The American Society of Human Genetics (ASHG) does not specifically address adult‐onset conditions but their general sentiment is that incidental findings of both childhood‐ and adult‐onset conditions should be reported, provided there is “clear clinical utility for the child and/or his or her family members.”^17^ In contrast, the CCMG suggests the general rule for such findings should be not to report, unless the result will be medically actionable for a family member, but only provided parents elected to receive this information prior to testing.^18^

These documents also differ in whether they allow the child's parents/legal guardians to choose whether they wish to receive incidental findings.^20^ Where some suggest parents should not be able to opt‐out of receiving incidental findings for serious and actionable conditions with childhood onset,^17^, ^18^ others allow parents to opt out.^5^

Regarding actively searching for secondary findings, whilst the ACMG advocate for these to be sought for all patients, regardless of age,^4^, ^5^, ^7^ the CCMG and ESHG state that this type of ‘opportunistic screening’ is not appropriate in children.^18^, ^19^

## 3 STAKEHOLDER PREFERENCES FOR, AND OUTCOMES FROM RETURNING SECONDARY FINDINGS

### 3.1 Healthcare professional perspectives

Few studies have examined health professionals' perspectives on returning secondary findings from prenatal GS.^21^, ^22^, ^23^ A survey of 498 members of the ACMG, ASHG and US National Society of Genetic Counselors, showed that 93% and 80% agreed that childhood‐onset conditions with and without treatment respectively should be returned to prospective parents.^22^ Yet participants were less positive about the return of results for both treatable (50%) and untreatable (34%) conditions with onset in adulthood. These participants felt that professional organizations should be responsible for determining which results are disclosed to families, rather than the healthcare provider or parents, although many (65%–66%) appeared to favor healthcare providers assisting parents to choose from either a full or limited set of return options. There was a 50/50 split on whether the existing ACMG guidelines should apply to the prenatal setting.

Focus groups with five patient group representatives and eight clinical professionals showed varying perspectives on return of incidental findings from prenatal GS.^23^ While some clinical professionals felt these should not be reported, others highlighted that this was potentially more appropriate when treatment options are available. On the other hand, patient representatives raised concerns about detrimental effects on the parent‐clinician relationship if incidental findings were initially withheld and then disclosed at a later date. Participants from both groups discussed concerns about the consent process, particularly in relation to the level of detail that needs to be covered regarding results unrelated to the initial reason for testing.

Conference attendees at the 2019 annual International Society of Prenatal Diagnosis (ISPD) meeting were polled before and after a debate on “Secondary findings in the 59 genes, that ACMG recommends reporting when sequencing postnatally, should also be reported when they are detected on fetal (and parental) samples during prenatal sequencing”.^24^ While participants initially favored reporting secondary findings (66%), at the conclusion of the debate this proportion had reduced to 55%. Finally, although a survey of 1114 obstetricians in the USA did not ask specifically about return of secondary findings, participants reported being concerned about an increase in parental anxiety in response to complex genomic information, and discomfort about communicating results to expectant parents.^21^

### 3.2 Parent perspectives

Several studies have explored parental preferences for receiving secondary findings from prenatal GS. Some of these studies report the proportion of parents who chose to receive secondary findings. For example, a summary of the experience of use of prenatal GS in Denmark from 2016 to 2019 explained that none of the 35 couples that received testing had chosen to opt out of receiving secondary findings, although their list of reported conditions did not correspond with that of the ACMG, choosing instead to report secondary findings they deemed relevant to the health of the fetus or future pregnancies.^25^ Similarly, a study in the USA performed a secondary analysis from two studies in which parents had been offered secondary findings prior to testing: one included prenatal cases with non‐immune hydrops and the other included both prenatal and pediatric samples.^26^ Of the 256 cases in the prenatal setting in which concordant preferences were expressed between both parents, 86.2% agreed to receive secondary findings.

Others have assessed parents' attitudes towards return of findings from prenatal GS.^27^, ^28^ One asked 186 expectant parents what information they would want to know from prenatal GS, although they did not frame this specifically as secondary findings.^27^ They found that most parents would want to receive information about childhood‐onset treatable (96%) and non‐treatable (86%) conditions. Agreement dropped for both treatable (76%) and non‐treatable (74%) conditions where onset was in adulthood, and also for variants of uncertain significance (71%). Another study interviewed 29 parents (17 of whom were women) who had already undergone prenatal GS for fetal abnormalities and asked what information they would have liked to receive.^28^ These parents expressed strong preferences to receive as much information as was available. The authors noted that if secondary findings were to be offered, more time would be required in consultations to accommodate the nuanced discussions required.

### 3.3 Disclosure outcomes

To our knowledge, only one study has explored the impact of prenatal GS on maternal psychological outcomes.^29^ The study enrolled 115 trios for prenatal GS to investigate an abnormal pregnancy, assessed the mothers on a range of psychological measures both pre‐ and post‐sequencing, and stratified them according to their result (64% received a negative result, 31% received a result that may explain the phenotype and 6% received secondary findings). Although women who received secondary findings reported higher generalized distress post‐sequencing, greater test‐related distress, and poorer psychological adaptation to testing than those who received a negative result, those who received a result that could explain the fetal phenotype showed higher scores across all these measures than both the negative and secondary findings groups. These women with potential explanations also showed increased depression compared to both other groups. In short, although women who receive secondary findings did experience some distress, they experienced less than those who receive a result related to the fetal phenotype.

## 4 PRACTICAL CONSIDERATIONS

Beyond the research setting, and the recommendations of expert bodies, a number of ‘real world’ challenges need to be considered in the context of returning incidental and secondary findings in the prenatal setting.

### 4.1 Test design

As noted above, the prevailing view is that a trio test design is ideal in the prenatal setting, allowing rapid detection of de novo variants and segregation of recessive variants, and reducing the reporting of uncertain variants.^11^, ^22^ The use of a trio design brings additional consent requirements for both parents, given that incidental or secondary findings may be detected in the parental as well as the fetal genotypes,^30^ and consanguinity and non‐paternity may also be detected.^31^

### 4.2 Which genes to report

If incidental and secondary findings are to be reported, decisions need to be made about which genes and which variants are reported. As a rule, only pathogenic and likely pathogenic variants are reported in the context of incidental and secondary findings, where there is no phenotype to guide interpretation, aligning with recommendations in the postnatal setting.^7^ Choice of genes is more complex. For incidental findings, there is no predefined gene list, and so each variant detected incidentally must be assessed individually against the criteria of disease severity, age of onset, and medical actionability.^11^ For secondary findings (SF), a starting point is the ACMG SF v3.0 gene list, which includes 73 genes, and which has been curated to maximize the potential to reduce morbidity and mortality, based on current standards of care.^8^ Most genes are linked to cancer or cardiovascular phenotypes, and some, but not all, are associated with childhood onset of symptoms. Importantly, the ACMG list has been recommended as a ‘minimum list’, leaving the opportunity open for laboratories to add more genes if relevant variants are both readily detectable and fulfill desirable criteria such as disease severity, age of onset, penetrance and actionability.^7^

A more expansive approach that could be applied in the prenatal setting would be to apply one of the customized gene lists that have been developed for genomic newborn screening.^32^, ^33^, ^34^ The gene list developed for the BabySeq project^32^ includes 954 genes and, in contrast to the ACMG SF v3.0 list, excludes adult onset disorders but includes carrier status. However, it includes childhood onset disorders for which there is no treatment, on the basis that early detection may eliminate the need for extensive clinical tests, reduce the diagnostic odyssey, and allow the implementation of non‐targeted early interventions that may improve outcomes. An important difference between newborn screening and prenatal diagnosis, however, is that in prenatal diagnosis a result may lead to a termination of pregnancy, which is contrary to the original conceptualization of ‘actionability’, where the primary purpose is to benefit the health of the individual being tested.^24^

### 4.3 Counselling and consent

It has been recommended that all testing for secondary findings be accompanied by pre‐ and post‐test counselling to discuss the types of possible results, limitations of testing, and medical implications of any result.^7^ The prenatal clinic is typically an emotionally charged environment where critical decisions are made within a short time frame and, as such, is not the ideal setting to counsel and obtain consent for secondary findings that may have distinct and longer‐term health implications for the family. Additional consent requirements represent a further burden to patients, and those delivering prenatal diagnosis services may lack the time and skills to counsel patients for a broad range of secondary findings, and to coordinate appropriate follow up when actionable findings are identified. In some circumstances it may be possible to conduct the pre‐test counselling and result disclosure for secondary findings at a separate time and location, for example through attendance at a specialized clinic that could take place at a time after the birth of the baby. Such a ‘two‐step’ approach to offering additional findings has been trialled in both the adult^35^ and newborn^36^ contexts, and may provide a solution to some of the challenges of offering secondary findings in the prenatal setting, alleviating time pressure and ensuring availability of suitably qualified staff.

We consider that analysis for secondary findings should never be performed automatically in the prenatal setting, but could be offered as either an opt‐in or –opt‐out option. Although the ACMG recommend that the option to opt‐out of secondary findings should be presented to the individual in the context of prenatal ES/GS,^7^ we consider that an opt‐in consent may be preferable, as recommended by the CCMG.^11^ This is because an opt‐out approach means that, if left unticked, analysis of secondary findings will occur by default when it is possible that the option was not discussed.^37^

A further consideration is how much choice should be offered regarding what secondary findings are returned. In the setting of prenatal CMA, it has been shown that people make different choices when offered different levels of genetic information^38^; yet whether they actually want to be offered choice is largely unexplored in the medical literature. Whilst offering choice could be viewed as enhancing autonomy, it may also represent an additional burden at what is already a vulnerable time, and too many options may undermine people's ability to make truly informed decisions.^39^ In addition, the offer of multiple, complex choices may not be practical, and may have undesirable outcomes, such as compromising laboratory turn‐around‐time and increasing the potential for error.

### 4.4 Laboratory workload

The final practical consideration involves the laboratory workload required to facilitate the analysis and reporting of secondary findings. Laboratories may either lack the additional resources to report secondary findings or impose additional charges for the further analysis and reporting required, and prenatal genetics services may not see there remit as including payment for these services. These issues highlight the important role of local laboratories and clinical service providers in deciding whether analysis of secondary finding is offered.

## 5 ETHICAL CONSIDERATIONS

There are also ethical challenges associated with deciding whether to return incidental and/or secondary findings.

Beauchamp and Childress describe four ethical principles that should be drawn on to guide medical practice: beneficence, nonmaleficence, autonomy and justice.^40^ Ethical challenges often arise where there is conflict between when attempting to promote these principles. In relation to returning incidental and/or secondary findings, several such conflicts arise.

For example, one could argue that promoting autonomy would involve allowing parents unrestricted choice whether or not they wish to receive incidental findings. This would mean that parents should be free to refuse information about severe, treatable childhood onset conditions that may arise in their child. However, by refusing this information, the parents are not only missing an opportunity to promote the wellbeing of their child (beneficence), but by denying their child an opportunity to access treatment they are inflicting harm and therefore not promoting nonmaleficence.

Likewise, one could argue that respecting autonomy means that parents have a right to receive information about their child's risk of developing a non‐treatable, adult‐onset condition such as Alzheimer disease. However, guidelines generally recommend that predictive testing of this nature should be delayed until the individual has capacity to make this decision for themselves.^41^ This is based on trying to both reduce the risk of psychosocial harm that this knowledge might have on the child and also protect the child's future autonomy to decide whether they wish to receive information of this nature. Of course, this is complicated by the fact that, in the prenatal setting, parents have the option to terminate the pregnancy.

Another ethical challenge relates to the concept of distributive justice. One of the arguments against offering secondary findings to adults and children is that only those in the population who are receiving diagnostic sequencing will have access to information to promote their health. Likewise, in the prenatal setting, only parents accessing sequencing for fetal abnormalities would be offered secondary findings. This is problematic because those in lower socioeconomic and rural areas often find it more difficult to access prenatal GS. As such, offering secondary findings may exacerbate existing health disparities unless we find ways to ensure fair and equitable access to this technology.

## 6 A FRAMEWORK FOR REPORTING INCIDENTAL AND SECONDARY FINDINGS IN PRENATAL SEQUENCING

Considering the arguments presented above, we propose a framework where a routine baseline analysis of genomic data would be limited to a phenotype‐driven interpretation. Reporting incidental findings would be limited to severe childhood onset disorders, and secondary findings neither sought not reported. Parental genomes, when available, would be analyzed only for the purpose of interpreting fetal findings, consistent with the primary purpose of the test.

Beyond this baseline routine analysis, additional layers of analysis could be offered, according to local laboratory policy, with additional opt‐in consent from the parents. Table 1 provides a schema for the layers of analysis that might be offered, acknowledging that this is not an exhaustive list of the combinations. According to this schema, additional levels of analysis could include: (1) extension of incidental findings to include adult‐onset disorders; (2) analysis for secondary findings in the fetus and parents, and (3) analysis of parental data for recessive carrier status. As noted earlier, the offer of too many choices could be harmful, and so it may be preferable to limit the number of choices offered. However, clinicians' concerns and patients' experiences do not always align, and potential for harm should not be assumed too readily at the expense of patient autonomy. Critically, implementation of this analysis should include close communication and cooperation between laboratories, service providers and patients.

**TABLE 1:** Framework phenotype‐specific gene panels or targeted interpretation of sequence data

	Level	Fetal analysis	Parental analysis	Incidental findings	Secondary findings
Routine analysis	1	Phenotype driven interpretation of sequence data	To interpret fetal findings only	Pediatric onset	None
Extended analysis according to local lab policy that is shared with patients and care provider. Only with additional opt‐in consent.	2a	Phenotype driven interpretation of sequence data	To interpret fetal findings only	Pediatric and adult onset	None
	2b	Phenotype driven interpretation of sequence data Plus secondary findings	To interpret fetal findings only	Pediatric and adult onset	Fetus only
	2c	Phenotype driven interpretation of sequence data Plus secondary findings	To interpret fetal findings Plus secondary findings	Pediatric and adult onset	Fetus and parents
	2d	Phenotype driven interpretation of sequence data Plus secondary findings	To interpret fetal findings. Plus secondary findings. Plus carrier status	Pediatric and adult onset	Fetus and parents Plus carrier status in parents

## 7 CONCLUDING COMMENTS

Reporting incidental findings from prenatal sequencing has the potential to provide information that can lead to prevention of potentially serious harm. Yet achievement of this goal depends on a number of interrelated factors that start with the sequencing technology itself, and extend through practical, ethical and counselling considerations, at all times taking into account the wants and needs of prospective parents. The framework suggested here will likely evolve over time, as much‐needed research provides empirical evidence that will, in time, replace expert opinion as the foundation of future recommendations.

## CONFLICT OF INTEREST

The authors declare no conflict of interest.

## Data Availability

The data that support the findings of this study are available from the corresponding author upon reasonable request.
